# Environmental Health Literacy and Climate Change Anxiety Among Teachers: The Mediating Role of Ecological Footprint Awareness

**DOI:** 10.3390/ijerph23050685

**Published:** 2026-05-21

**Authors:** Özge Açıkgöz, Pınar Soylar

**Affiliations:** Department of Nursing, Health Sciences Faculty, Fırat University, Elazig 23119, Türkiye

**Keywords:** environmental health literacy, ecological footprint awareness, climate change anxiety, teachers, mediation analysis

## Abstract

**Highlights:**

**Public health relevance—How does this work relate to a public health issue?**

- Climate change anxiety and environmental awareness are emerging public health concerns affecting psychological well-being.
- Understanding the role of environmental health literacy in shaping climate-related emotional responses is essential for promoting sustainable behaviors.

**Public health significance—Why is this work of significance to public health?**

- This study identifies ecological footprint awareness as a key mechanism linking environmental health literacy to climate change anxiety.
- The findings contribute to the limited evidence on how environmental knowledge translates into psychological and behavioral responses in public health contexts.

**Public health implications—What are the key implications or messages for practitioners, policymakers and/or researchers in public health?**

- Environmental education programs should integrate ecological footprint-based approaches to strengthen both awareness and emotional engagement with climate issues.
- Teachers can play a critical role in promoting sustainable behaviors and climate awareness, supporting long-term public health and environmental outcomes.

**Abstract:**

Background: Environmental health literacy plays an important role in helping individuals recognize environmental risks and adopt sustainable behaviors. Increasing environmental awareness may also influence emotional responses to environmental problems such as climate change. However, the mechanisms linking environmental health literacy to climate change anxiety remain insufficiently explored. This study aimed to examine the relationship between environmental health literacy and climate change anxiety among teachers and to evaluate the mediating role of ecological footprint awareness in this relationship. Methods: This cross-sectional study was conducted with teachers working in public schools in the provinces of Elazığ and Erzincan, Türkiye. Data were collected using a personal information form, the Environmental Health Literacy Scale, the Ecological Footprint Awareness Scale, and the Climate Change Anxiety Scale. Descriptive statistics, group comparison tests, correlation analyses, and mediation analysis based on structural equation modeling were performed to examine the relationships among the study variables. Results: Participants’ mean scores were 35.98 ± 9.12 for the Climate Change Anxiety Scale, 95.37 ± 18.29 for the Environmental Health Literacy Scale, and 118.08 ± 25.92 for the Ecological Footprint Awareness Scale. Environmental health literacy was positively associated with ecological footprint awareness, and ecological footprint awareness was positively associated with climate change anxiety (*p* < 0.001). Mediation analysis indicated that ecological footprint awareness significantly mediated the relationship between environmental health literacy and climate change anxiety (β = 0.293, 95% CI: 0.112–0.496, *p* = 0.002). Conclusions: The findings suggest that ecological awareness can serve as a potential mechanism linking environmental knowledge with emotional responses to climate change. Strengthening ecological footprint awareness through environmental education programs for teachers may contribute to both environmental awareness and constructive engagement with climate-related issues.

## 1. Introduction

In today’s world, environmental problems such as climate change, drought, desertification, deforestation, and marine and ocean pollution are becoming increasingly visible, posing an unprecedented threat to future generations [1,2]. According to the World Health Organization (WHO), approximately one-quarter (around 24%) of global deaths are still attributable to modifiable environmental risks, highlighting the persistent and substantial impact of environmental factors on population health [3]. The major health conditions responsible for these environmentally related deaths include circulatory system diseases, cancers, chronic respiratory diseases, infectious diseases, and injuries [4,5]. Similarly, environmental exposures such as air pollution, hazardous chemicals, and ultraviolet radiation are responsible for a substantial proportion of the disease burden in Europe [6].

Although the impacts of climate change have traditionally been examined in terms of physical health outcomes, growing evidence suggests that environmental degradation also affects mental health and psychosocial well-being [7,8]. In this context, climate change anxiety has emerged as an important psychological response, defined as the intense worry and concern experienced regarding potential environmental threats and future uncertainties [9]. Climate change anxiety refers to the emotional distress, worry, or concern individuals experience in response to the perceived threats of climate change and environmental uncertainty [9,10,11,12,13].

Environmental health literacy refers to individuals’ ability to access, understand, and use information about environmental health risks in order to make informed decisions and adopt behaviors that protect both individual and public health [14,15]. Increasing environmental health literacy contributes to greater awareness of environmental problems and supports the development of protective and sustainable behaviors. In this context, ecological footprint awareness refers to individuals’ understanding of how their daily consumption patterns and lifestyle choices affect the environment and natural resources [15,16]. As awareness of environmental impacts increases, individuals may also become more sensitive to environmental threats such as climate change.

The concept of sustainability aims to ensure the fair and balanced use of resources across economic, social, and ecological dimensions across generations, allowing future generations to benefit from resources in a manner comparable to the present generation. In this context, ecological footprint awareness is considered an important tool that helps individuals understand the environmental impacts of their consumption habits and adopt more sustainable behaviors [16]. Environmental problems arising from individual and societal consumption patterns are among the main drivers of climate change on a global scale [17,18]. In this regard, increasing environmental awareness enhances individuals’ sensitivity to environmental issues and contributes to the development of a culture of sustainability, particularly within educational settings. Ecological footprint awareness makes the relationship between daily life practices and environmental impacts more visible and is considered an important cognitive and behavioral mechanism that may motivate individuals to adopt more sustainable consumption patterns [16].

Teachers play a strategic role in promoting public health and environmental awareness, as they influence students’ knowledge, attitudes, and behaviors and contribute to broader societal outcomes [15,19]. Previous research has emphasized that teachers are key agents in fostering environmental literacy and sustainability-related behaviors among younger generations. In addition, teachers serve as role models within educational settings, and their level of environmental awareness can directly influence students’ perceptions and long-term behavioral patterns [14,15,17,19]. Therefore, examining environmental health literacy and related psychological responses among teachers is essential for understanding how environmental knowledge and awareness may be transmitted to the wider community. Despite their critical role, previous studies have generally examined environmental health literacy, ecological awareness, and climate change anxiety as separate constructs, while research investigating the mechanisms linking these variables remains limited. While previous studies have examined these constructs, the novelty of this study lies in investigating ecological footprint awareness as a mediating mechanism, particularly within a sample of teachers. The present study was conducted among teachers working in public schools in Elazığ and Erzincan, Türkiye. Teachers were selected as the study population due to their strategic role in promoting environmental awareness and sustainable behaviors within the community. In addition, regional and contextual differences may influence environmental awareness and perceptions of climate change, making it important to examine these relationships within a specific national context. Based on this framework, this study aimed to examine the relationship between environmental health literacy and climate change anxiety among teachers and to evaluate the mediating role of ecological footprint awareness in this relationship. The following hypotheses were tested:

**H1.**  *Environmental health literacy is significantly associated with ecological footprint awareness among teachers.*

**H2.**  *Ecological footprint awareness statistically accounts for the relationship between environmental health literacy and climate change anxiety among teachers.*

## 2. Methods

### 2.1. Study Design

**Figure 1:**
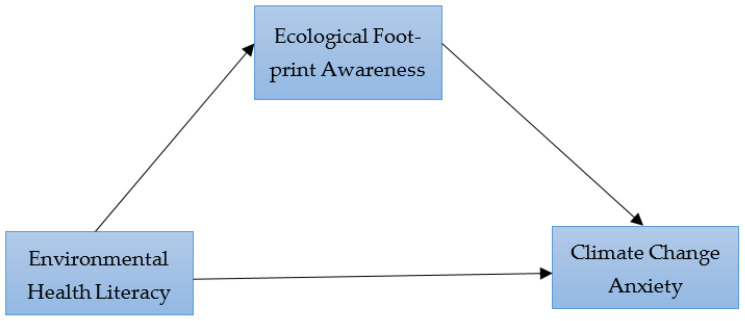
Conceptual model of the study showing the hypothesized relationships between environmental health literacy, ecological footprint awareness, and climate change anxiety.

This study employed a quantitative cross-sectional design, which involves the collection of data at a single point in time to examine relationships among variables without implying causality [20,21]. Despite its limitations, cross-sectional designs continue to be widely used in contemporary health and behavioral research (e.g., recent studies published after 2022), supporting their ongoing relevance in the field. Conceptual model illustrating the mediating role of ecological footprint awareness in the relationship between environmental health literacy and climate change anxiety (Figure 1).

### 2.2. Study Setting

**Figure 2:**
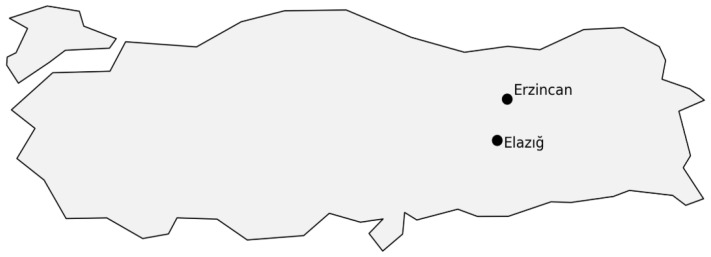
Geographic location of the study area showing the provinces of Elazığ and Erzincan in Türkiye.

The study was conducted in public primary, secondary, and high schools affiliated with the Turkish Ministry of National Education in the provinces of Erzincan and Elazığ, Türkiye. Data were collected between 30 April and 17 September 2025, during the active academic period. Both face-to-face and online data collection methods were used. The geographical locations of the study areas (Elazığ and Erzincan) within Türkiye are presented in Figure 2.

### 2.3. Population and Sample

The study population consisted of teachers working in public primary, secondary, and high schools in Erzincan and Elazığ during the study period. The required sample size was calculated using G*Power 3.1 based on the correlation coefficient reported by Yıldız et al. (2023) between environmental literacy and ecological footprint awareness (r = 0.234) [22]. With α = 0.05, power (1 − β) = 0.95, and effect size = 0.234, the minimum sample size was calculated as 193 participants.

A stratified sampling method was used to ensure proportional representation of teachers from different school levels (primary, secondary, and high school). The total number of teachers in the two provinces was 12,380 (Erzincan: 3637; Elazığ: 8743). Accordingly, the planned sample distribution was 29.4% from Erzincan (*n* ≈ 57) and 70.6% from Elazığ (n ≈ 136).

Because mediation analyses require a sufficient sample size to estimate indirect effects more reliably, data collection was extended to improve the precision and statistical power of the analysis [23]. As a result, data were collected from 281 teachers. The sampling process aimed to ensure proportional representation of teachers from different provinces, with approximately 74% of participants from Elazığ and 26% from Erzincan, reflecting the distribution of the target population.

### 2.4. Inclusion Criteria

Participants were included in the study if they met the following criteria:

- Voluntarily agreed to participate in the research;
- Had no communication difficulties or psychological conditions that could hinder participation;
- Were employed as teachers in schools affiliated with the Ministry of National Education in Erzincan or Elazığ.

### 2.5. Data Collection Instruments

The questionnaire was based on a theoretically informed model that assumes environmental health literacy may be associated with emotional responses to climate change, both directly and indirectly through ecological footprint awareness. Ecological footprint awareness refers to individuals’ understanding of the environmental impacts of their consumption patterns and their ability to evaluate these impacts in terms of sustainability [24].

### 2.6. Demographic Information Form

A demographic questionnaire consisting of nine items was developed to obtain information on teachers’ sociodemographic and professional characteristics. The form was prepared based on a review of the relevant literature and similar studies, and its content was evaluated for clarity and relevance. The questionnaire included items on age, gender, education level, teaching branch, school type, professional experience, city of residence, type of settlement primarily lived in, and whether the participant had previously received environmental health education.

### 2.7. Climate Change Anxiety Scale

The Climate Change Anxiety Scale, originally developed by Stewart (2021) and adapted into Turkish by Özbay and Alcı (2021), was used to measure individuals’ anxiety related to climate change [25,26]. The scale consists of 10 items and a single dimension rated on a 5-point Likert scale ranging from 1 (Never) to 5 (Always). Total scores range from 10 to 50, with higher scores indicating greater climate change anxiety. The Cronbach’s alpha coefficient was reported as 0.98 in the Turkish validation study and 0.95 in the present study [25,26]. The continued use of this scale in recent research supports its validity and applicability in assessing climate-related emotional responses.

### 2.8. Environmental Health Literacy Scale

Environmental health literacy was measured using the Environmental Health Literacy Scale developed by Toplu (2023) [27]. The scale contains 23 items and four subdimensions: Knowledge (items 1–6), Awareness of Health Effects (items 7–11), Perception of Environmental Exposure (items 12–15), and Behavior (items 16–23). Items 12–15 are reverse-scored. Responses are rated on a 5-point Likert scale ranging from 1 (Strongly disagree) to 5 (Strongly agree). Total scores range from 23 to 115, with higher scores indicating higher environmental health literacy. Cronbach’s alpha was 0.85 in the original study and 0.95 in the present study [27].

### 2.9. Ecological Footprint Awareness Scale

Ecological footprint awareness was assessed using the Ecological Footprint Awareness Scale developed by Tekindal et al. (2021) [28]. The scale consists of 30 items rated on a 5-point Likert scale (1 = Strongly disagree to 5 = Strongly agree) and includes six subdimensions: Energy, Legal Framework, Recycling, Transportation, Food, and Water Consumption. Total scores range from 30 to 150, with higher scores indicating greater ecological footprint awareness. In this study, analyses were conducted using the total scale score rather than subdimension scores. The Cronbach’s alpha coefficient was 0.96 in the original study and 0.97 in the current study [28]. The continued use of ecological footprint-based measures in recent research supports the relevance of this scale for assessing sustainability-related awareness.

### 2.10. Data Collection Procedure

Ethical approval for the study was obtained from the Non-Interventional Research Ethics Committee of Fırat University (Decision No: 2025/01-15, dated 9 January 2025). After obtaining the necessary institutional permissions, data were collected using the demographic information form, Climate Change Anxiety Scale, Environmental Health Literacy Scale, and Ecological Footprint Awareness Scale.

School principals were first contacted to provide information about the study and obtain permission. Subsequently, an online survey form created via Google Forms was distributed to teachers through school communication groups with the support of school administrators. To facilitate participation and increase response rates, some data were also collected through face-to-face administration. The average completion time for the questionnaire was approximately 10–15 min.

### 2.11. Statistical Analysis

All data were analyzed using SPSS for Windows version 22.0. Descriptive statistics are presented as numbers, percentages, minimum and maximum values, means, and standard deviations. The normality of the data distribution was evaluated using skewness and kurtosis coefficients, with values between ±2 considered acceptable.

For comparisons between two independent groups, the independent-samples *t*-test was used for normally distributed variables and the Mann–Whitney U test for non-normally distributed variables. For comparisons among more than two groups, one-way analysis of variance (ANOVA) or the Kruskal–Wallis test was applied as appropriate. Post hoc analyses were conducted using LSD or Dunnett’s C tests for ANOVA and Mann–Whitney U tests following the Kruskal–Wallis test. Relationships between variables were assessed using Spearman correlation analyses, depending on the distribution of the data. The internal consistency of the scales was evaluated using Cronbach’s alpha coefficient. The high Cronbach’s alpha value for the ecological footprint awareness scale may indicate strong internal consistency; however, it may also reflect item redundancy, which should be considered in future research. In this study, analyses were conducted using total scale scores to provide an overall assessment of the constructs and to maintain consistency with the mediation model. Subdimensions were not analyzed separately to avoid model complexity and potential multicollinearity issues.

To examine the hypothesized relationships among environmental health literacy, ecological footprint awareness, and climate change anxiety, a regression-based mediation (path) analysis was used. In this framework, environmental health literacy was treated as the independent variable, climate change anxiety as the dependent variable, and ecological footprint awareness as the mediator. The analysis involved estimating the direct effect (c′ path), the indirect effect (a × b path), and the total effect (c path). The significance of indirect effects was tested using bootstrapping with 5000 resamples, following established procedures for mediation analysis [23,29,30]. An indirect effect was considered statistically significant when the 95% confidence interval did not include zero. Standardized regression coefficients (β) are reported to indicate the strength and direction of relationships among variables. The model was interpreted based on the statistical significance of the path coefficients (*p* < 0.05) and the confidence intervals of the indirect effects.

## 3. Results

The findings related to climate change anxiety, environmental health literacy, and ecological footprint awareness among teachers, as well as the relationships and mediation effects among these variables, are presented below.

As presented in Table 1, the mean score of the participants was 35.98 ± 9.12 for the Climate Change Anxiety Scale, 95.37 ± 18.29 for the Environmental Health Literacy Scale, and 118.08 ± 25.92 for the Ecological Footprint Awareness Scale.

Table 2 presents the comparison of climate change anxiety, environmental health literacy, and ecological footprint awareness scores according to the demographic characteristics of the teachers. Climate change anxiety scores did not differ significantly according to age, gender, education level, teaching subject, years of professional experience, school type, or previous environmental health training (*p* > 0.05). Similarly, environmental health literacy scores were not significantly associated with these demographic variables (*p* > 0.05). Ecological footprint awareness scores also showed no significant differences according to most demographic characteristics (*p* > 0.05). However, ecological footprint awareness differed significantly according to teaching subject (*p* = 0.040) and previous environmental health training (*p* = 0.021). Teachers who had received prior environmental health training demonstrated higher ecological footprint awareness scores compared to those who had not received such training.

Spearman correlation analysis indicated significant positive associations among the study variables (Table 3). Climate change anxiety was positively associated with environmental health literacy (r = 0.246, *p* < 0.001) and ecological footprint awareness (r = 0.349, *p* < 0.001). Environmental health literacy was also positively and moderately correlated with ecological footprint awareness (r = 0.564, *p* < 0.001).

**Figure 3:**
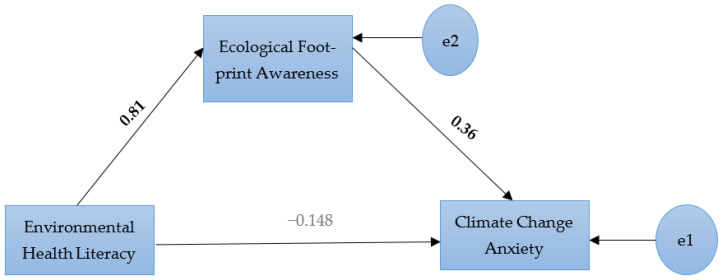
Structural equation model showing the mediating role of ecological footprint awareness in the relationship between environmental health literacy and climate change anxiety. e1 and e2 represent residual/error terms.

A regression-based mediation (path) analysis was conducted to examine the mediating role of ecological footprint awareness in the relationship between environmental health literacy and climate change anxiety. Because the model included a limited number of observed variables, it represents a saturated model; therefore, model fit indices were not considered necessary. The path coefficients for the direct, indirect, and total effects are presented in Table 4. Environmental health literacy had a significant positive effect on ecological footprint awareness (β = 0.810, *p* < 0.001). Ecological footprint awareness was positively associated with climate change anxiety (β = 0.362, *p* = 0.002). However, the direct effect of environmental health literacy on climate change anxiety was not statistically significant (β = −0.148, *p* = 0.165). The indirect effect of environmental health literacy on climate change anxiety through ecological footprint awareness was significant (β = 0.293, *p* = 0.002), indicating a mediating effect. In addition, the total effect of environmental health literacy on climate change anxiety was statistically significant (β = 0.144, *p* = 0.025). The structural equation model illustrating the mediating role of ecological footprint awareness is presented in Figure 3.

## 4. Discussion

The present study investigated the relationship between environmental health literacy and climate change anxiety among teachers and examined the mediating role of ecological footprint awareness in this relationship. The findings revealed that ecological footprint awareness differed according to certain professional characteristics and environmental education status. In addition, environmental health literacy was positively associated with ecological footprint awareness, and ecological footprint awareness was positively related to climate change anxiety. Importantly, the mediation analysis indicated that environmental health literacy did not directly affect climate change anxiety but influenced it indirectly through ecological footprint awareness. These findings suggest that ecological awareness may play a key role in linking environmental knowledge with emotional responses to climate change.

Regarding demographic variables, ecological footprint awareness did not differ significantly according to gender. This finding is consistent with previous studies reporting no gender differences in ecological footprint awareness [31,32,33]. However, ecological footprint awareness differed significantly according to teaching subject and environmental education status. Classroom teachers showed the highest levels of ecological footprint awareness, followed by Turkish language and science teachers, whereas foreign language teachers had the lowest scores. Similar findings were reported by Demirkol and Aslan (2021), who found relatively high ecological awareness among classroom teachers [31,34]. Nevertheless, some studies conducted with teacher candidates have reported higher ecological footprint awareness among science teacher candidates compared with classroom teacher candidates [35,36]. This discrepancy may be related to differences in the study samples and educational contexts. Unlike many previous studies that focused on teacher candidates within a single educational level, the present study included teachers working at different school levels. Differences in professional experience, teaching environments, and the scope of environmental topics within curricula may therefore explain these variations. This difference may also reflect variations in exposure to environmental content and professional training opportunities across disciplines.

In addition, teachers who had received environmental education demonstrated significantly higher ecological footprint awareness compared with those who had not received such training. Similar results have been reported in previous studies showing that environmental education increases ecological awareness and environmentally responsible behaviors [36,37]. Likewise, studies have shown that students who receive education related to environmental and climate issues exhibit higher ecological awareness compared with those who do not receive such education [38,39]. These findings suggest that increasing environmental knowledge may contribute to greater ecological awareness.

Another important finding of the study was the positive relationship between environmental health literacy and ecological footprint awareness. Teachers with higher environmental health literacy levels also demonstrated greater ecological footprint awareness. This finding is consistent with previous research indicating that environmental literacy contributes to environmental awareness and pro-environmental attitudes. For example, Eren et al. (2025) found a direct relationship between environmental health literacy and environmental attitudes among pharmacy students [40]. Similarly, Yıldırım et al. (2025) reported that environmental literacy, ecological footprint awareness, and environmental behavior were positively related among adults [41]. These findings indicate that improving environmental literacy may play a key role in enhancing ecological awareness and encouraging sustainable behaviors. For example, individuals with higher ecological footprint awareness may be more likely to prefer reusable products instead of single-use materials, reduce unnecessary consumption, or adopt energy-saving habits in their daily lives.

The results also revealed a significant positive relationship between ecological footprint awareness and climate change anxiety. Individuals with higher levels of ecological awareness may be more sensitive to the potential consequences of climate change and therefore experience greater concern regarding environmental problems. Similar findings have been reported in previous research. For example, Ergun et al. (2021) found that individuals living in rural areas exhibited higher levels of climate change concern than those living in urban areas, suggesting that increased environmental awareness may contribute to higher levels of climate-related concern [42]. Likewise, Bouman et al. (2020) reported that climate concern can promote support for climate policies and environmentally responsible behaviors, largely through individuals’ sense of personal responsibility [43]. More recent research also supports this relationship, indicating that climate-related emotional responses are associated with increased environmental awareness and behavioral engagement, while also being linked to reduced psychological well-being [44].

However, some studies have suggested an alternative direction for this relationship, indicating that climate change anxiety may increase environmental awareness rather than the reverse [45,46,47,48]. For example, Aman et al. (2021) found that tourists with higher environmental awareness experienced greater concern about environmental problems [45]. Similarly, Yılmaz et al. (2018) reported that increased knowledge about climate change increased climate anxiety, which subsequently contributed to the development of environmental awareness [47]. Conversely, other studies have reported no significant relationship between climate change awareness and climate anxiety [48]. These mixed findings suggest that the relationship between awareness and anxiety may vary depending on contextual factors, sample characteristics, and measurement approaches.

Finally, the mediation analysis demonstrated that ecological footprint awareness plays a mediating role in the relationship between environmental health literacy and climate change anxiety. Environmental health literacy did not have a direct effect on climate change anxiety; however, it influenced climate change anxiety indirectly through ecological footprint awareness. The pattern of results, characterized by a non-significant negative direct effect alongside a significant positive indirect and total effect, may indicate a suppression effect. This suggests that ecological footprint awareness may reveal underlying relationships that are not directly observable in the bivariate association. This finding suggests that individuals with higher environmental health literacy may become more aware of the environmental consequences of their behaviors, which in turn increases their concern about climate change. To the best of our knowledge, studies examining these three variables together remain limited, and therefore, the present study contributes to the literature by highlighting the mediating role of ecological awareness in this relationship.

The findings of the present study may have potential implications for environmental education and public health strategies. Educational programs addressing environmental and climate issues can be structured to include both knowledge-based and behavior-oriented components. For example, such programs may incorporate interactive learning methods, case-based discussions on climate-related health risks, and problem-solving activities that encourage students to reflect on real-life environmental challenges. Integrating topics such as sustainable consumption, waste management, and climate-related health impacts into school curricula may further enhance environmental awareness and engagement. In addition, activities based on ecological footprint awareness can provide practical opportunities for individuals to connect their daily behaviors with environmental outcomes. However, it is important to consider that the climate change anxiety scale used in this study reflects maladaptive aspects of emotional responses, such as distress and functional impairment (e.g., sleep disturbances or excessive worry). Therefore, increasing ecological footprint awareness may not necessarily lead to positive engagement, but it may also intensify emotional distress in some individuals. This highlights the need for carefully designed educational interventions that balance awareness with psychological resilience.

These activities may include calculating personal ecological footprints, monitoring resource use (such as water and energy consumption), organizing recycling initiatives, and promoting the use of reusable materials. Such practices can help individuals better understand the environmental consequences of their actions and support the development of more sustainable habits.

This study has several limitations that should be considered when interpreting the findings. First, the data were collected from teachers working in the provinces of Elazığ and Erzincan, which may limit the generalizability of the results to the broader population of teachers in Türkiye [20,21]. Second, the study was conducted within a specific time period; therefore, the findings may not fully reflect changes in environmental awareness or climate change anxiety over time, as cross-sectional designs do not allow for the examination of temporal relationships or causal inferences [20]. In addition, the use of both online and face-to-face data collection methods may have introduced variability in responses. Finally, the data were based on participants’ self-reports, which may be subject to response bias or social desirability bias, common limitations in survey-based research that may affect the accuracy of the findings [49,50,51].

To address these limitations, future studies should consider including more diverse and representative samples from different regions and educational contexts to improve generalizability. Longitudinal research designs may provide a better understanding of how environmental health literacy, ecological footprint awareness, and climate change anxiety evolve over time and allow for stronger inferences regarding potential causal relationships. Furthermore, the use of mixed-methods approaches, including qualitative data collection techniques such as interviews or focus groups, may help to gain deeper insights into individuals’ perceptions and emotional responses to environmental issues. To reduce potential response and social desirability bias, future studies may also incorporate objective or behavioral measures, such as observations or ecological footprint tracking tools, alongside self-reported data.

## 5. Conclusions

This study examined the relationship between environmental health literacy and climate change anxiety among teachers and evaluated the mediating role of ecological footprint awareness. The findings partially supported the proposed framework. The findings indicate that environmental health literacy is positively associated with ecological footprint awareness, and ecological footprint awareness is positively related to climate change anxiety. However, environmental health literacy did not directly affect climate change anxiety; instead, its effect occurred indirectly through ecological footprint awareness. Accordingly, H1 was supported, demonstrating a significant relationship between environmental health literacy and ecological footprint awareness. H2 was also supported, with findings indicating that ecological footprint awareness mediates the relationship between environmental health literacy and climate change anxiety.

These findings suggest that ecological awareness may be linked to emotional responses to climate change; however, such responses should be interpreted with caution. In this study, climate change anxiety reflects maladaptive aspects of emotional responses, such as distress and functional impairment, rather than necessarily indicating constructive engagement. It is therefore important to distinguish between adaptive forms of emotional response, such as concern, and maladaptive responses, such as anxiety. From a policy perspective, environmental education programs should not only aim to increase environmental knowledge and awareness but also consider individuals’ psychological responses. Interventions that promote environmental awareness may benefit from incorporating components that support coping strategies and psychological resilience. Finally, the findings should be interpreted in light of certain limitations. Due to the cross-sectional design, causal inferences cannot be made, and the results are limited to the specific sample of teachers included in this study, which may restrict generalizability.

## Figures and Tables

**Table 1:** Distribution of scores on the Climate Change Anxiety Scale, Ecological Footprint Awareness Scale, and Environmental Health Literacy Scale.

Scales	n	Min	Max	Mean	SD
Climate Change Anxiety Scale	281	10.00	50.00	35.98	9.12
Environmental Health Literacy Scale	281	35.00	115.00	95.37	18.29
Ecological Footprint Awareness Scale	281	30.00	150.00	118.08	25.92

**Table 2:** Comparison of climate change anxiety, environmental health literacy, and ecological footprint awareness scores according to demographic characteristics.

		n	Climate Change Anxiety	Environmental Health Literacy	Ecological Footprint Awareness
			Mean ± SD	Mean ± SD	Mean ± SD
Age	21–30 years	50	34.42 ± 8.74	95.30 ± 11.97	117.24 ± 19.42
	31–40 years	88	36.34 ± 9.16	95.66 ± 18.20	117.65 ± 24.23
	41–50 years	88	35.08 ± 9.78	97.03 ± 16.91	120.49 ± 25.53
	51–60 years and above	55	38.24 ± 7.98	92.33 ± 24.42	115.69 ± 33.61
	p	F = 1.961 0.120		*x*^2^_KW_ = 4.715 0.194	*x*^2^_KW_ = 4.540 0.209
Gender	Female	139	36.52 ± 8.48	95.99 ± 17.33	118.53 ± 26.06
	male	142	35.44 ± 9.71	94.77 ± 19.22	117.64 ± 25.87
	p	t = 0.987 0.324		U = 9.757.500 0.870	U = 9.740.000 0.850
Education Level	Bachelor’s degree	226	35.94 ± 9.21	95.87 ± 17.66	118.18 ± 26.01
	Master’s or doctoral degree	55	36.11 ± 8.84	93.33 ± 20.33	117.69 ± 25.77
	p	t = −0.121 0.904		U = 5.785.500 0.462	U = 6.140.000 0.890
Teaching subject of teachers	Classroom teachers	71	37.25 ± 7.39	96.31 ± 17.32	121.75 ± 26.25
	Physical education/visual arts/music teachers	33	36.12 ± 9.03	93.42 ± 21.79	114.42 ± 28.22
	Foreign language teachers	18	37.67 ± 9.83	89.50 ± 24.35	112.39 ± 34.59
	Social sciences teachers	59	34.69 ± 11.29	96.10 ± 16.07	118.12 ± 21.85
	Science teachers	54	37.48 ± 8.54	95.35 ± 17.76	118.78 ± 25.07
	Mathematics teachers	25	32.88 ± 8.55	96.80 ± 15.43	112.48 ± 20.53
	Turkish language and literature teachers	21	33.38 ± 8.46	96.62 ± 21.30	121.10 ± 31.56
	p	F = 1.558 0.160		*x*^2^_KW_ = 3.073 0.800	*x*^2^_KW_ = 13.186 0.040
Years of professional experience	0–5 years	52	35.19 ± 8.57	96.04 ± 12.31	120.50 ± 15.39
	6–10 years	37	36.49 ± 8.23	94.95 ± 19.26	113.05 ± 28.37
	11–15 years	53	34.26 ± 10.31	92.79 ± 20.89	113.85 ± 29.55
	16–20 years	42	38.43 ± 7.81	98.83 ± 15.85	122.33 ± 23.35
	≥21 years	97	36.07 ± 9.48	95.09 ± 20.08	119.18 ± 28.18
	p	F = 0.769 0.465		*x*^2^_KW_ = 5.314 0.257	*x*^2^_KW_ = 7.446 0.114
Type of school	Primary School	88	36.94 ± 8.37	95.69 ± 17.33	120.49 ± 26.25
	Secondary School	87	35.76 ± 9.21	93.03 ± 21.29	113.86 ± 28.43
	High School	106	35.35 ± 9.65	97.03 ± 16.27	119.55 ± 23.17
	p	F = 0.769 0.465		*x*^2^_KW_ = 0.936 0.626	*x*^2^_KW_ = 4.403 0.111
Previous training on environmental health	Yes	95	36.64 ± 8.23	96.06 ± 18.02	121.29 ± 24.94
	No	186	35.63 ± 9.55	95.02 ± 18.46	116.44 ± 26.32
	p	t = 0.876 0.382		U = 8.508.000 0.612	U = 7.350.500 0.021

t: independent-samples *t*-test; F: analysis of variance (ANOVA); U: Mann–Whitney U test; χ^2^KW: Kruskal–Wallis test.

**Table 3:** Relationships among climate change anxiety, ecological footprint awareness, and environmental health literacy.

		Climate Change Anxiety	Environmental Health Literacy	Ecological Footprint Awareness
Climate Change Anxiety	r *	1.000	0.246	0.349
	*p*	.	<0.001	<0.001
	*n*	281	281	281
Environmental Health Literacy	*r*	0.246	1.000	0.564
	*p*	<0.001	.	<0.001
	*n*	281	281	281
Ecological Footprint Awareness	*r*	0.349	0.564	1.000
	*p*	<0.001	<0.001	.
	*n*	281	281	281

* Spearman correlation coefficients are presented. “.” indicates that *p*-values were not calculated for correlations of variables with themselves.

**Table 4:** Path coefficients for the direct, indirect, and total effects of the model.

Path	*β*	*p*	95% CI	
			Lower	Upper
Environmental Health Literacy → Ecological Footprint Awareness (Direct effect, path a)	0.810	0.001	0.710	0.875
Ecological Footprint Awareness → Climate Change Anxiety (Direct effect, path b)	0.362	0.002	0.134	0.577
Environmental Health Literacy → Climate Change Anxiety (Direct effect, path c’)	−0.148	0.165	−0.385	0.075
Environmental Health Literacy → Climate Change Anxiety (Indirect effect, path a × b)	0.293	0.002	0.112	0.496
Environmental Health Literacy → Climate Change Anxiety (Total effect, path c)	0.144	0.025	0.019	0.278

Path a: effect of environmental health literacy on ecological footprint awareness. Path b: effect of ecological footprint awareness on climate change anxiety. Path c’: direct effect of environmental health literacy on climate change anxiety. Path a × b: indirect effect. Path c: total effect.

## Data Availability

The datasets used and/or analyzed during the current study are available from the corresponding author upon reasonable request.
